# Relative Entropy Method Applied for the Fatigue Life Distribution of Carbon Fiber/Epoxy Composites

**DOI:** 10.3390/e23020224

**Published:** 2021-02-11

**Authors:** Changsheng Yuan, Yingjie Liang

**Affiliations:** Institute of Soft Matter Mechanics, College of Mechanics and Materials, Hohai University, Nanjing 211100, China; yuanchangs@hhu.edu.cn

**Keywords:** fatigue life, Mittag-Leffler distribution, relative entropy, fractional order moment, logarithmic moment, carbon fiber/epoxy composites

## Abstract

This paper verifies the feasibility of the relative entropy method in selecting the most suitable statistical distribution for the experimental data, which do not follow an exponential distribution. The efficiency of the relative entropy method is tested through the fractional order moment and the logarithmic moment in terms of the experimental data of carbon fiber/epoxy composites with different stress amplitudes. For better usage of the relative entropy method, the efficient range of its application is also studied. The application results show that the relative entropy method is not very fit for choosing the proper distribution for non-exponential random data when the heavy tail trait of the experimental data is emphasized. It is not consistent with the Kolmogorov–Smirnov test but is consistent with the residual sum of squares in the least squares method whenever it is calculated by the fractional moment or the logarithmic moment. Under different stress amplitudes, the relative entropy method has different performances.

## 1. Introduction

Study on the fatigue of materials has become more and more significant since Wilhelm Albert published the first article on fatigue in 1837 [1,2]. At the same time, engineering application needs more diverse materials, especially composite materials, and requires some materials working under extreme conditions, such as high-speed railways and aerospace [3,4]. Thus, the importance of the fatigue of a material is beyond just an academic interest and comes into the field of real practice. However, essentially, the fatigue of a material is a statistical problem in the sense that if we want to predicate its behavior, then we should allow some intrinsic uncertainty. Except for the uncertainty of working conditions and measurement, the intrinsic uncertainty origin from dynamic chaos should also be considered [5,6,7,8]. Thus, a few probability and statistic theory tools are selected to describe the behavior of fatigue for different kinds of materials, such as the frequently used exponential distribution and the Weibull distribution [9,10,11].

It is known that the exponential distribution was the to be first studied completely, then the Weibull distribution became popular in predication of longevity and reliability. The phenomenon follows non-exponential distributions, which are ignored or too difficult to be analyzed, and is receiving more attention from both academia and industry [12,13,14]. Just as nonlinearity is after linearity, nonequilibrium is after equilibrium and noncommutative is after commutative, being a “negation” of the preceding classic theory (with non- as a prefix), non-exponential distribution shares the common hard nuts to crack or tasks with this “post-theory” or “meta-theory”. The first problem is how to accurately depict non-exponential phenomena; the second is how to deal with the explosive growth of the computational cost, with even singularities being hard to be removed [15,16].

Non-exponential distributions are superior to exponential ones for their good performances in various fields dealing with complex systems such as finance, life and climate [12]. Specifically, the Mittag-Leffler (M-L) distribution is accepted as the proper method to study the fatigue of composites. In this study, we would like to investigate the feasibility of the M-L distribution in fitting the fatigue data based on the relative entropy method [17]. Nowadays, the M-L distribution has been applied as a novelty statistical tool to describe non-exponential statistical phenomena in diverse fields [18,19], such as bridge fatigue life assessment [12] and modeling of an anomalous diffusion with hereditary effects for the importance of the M-L function in the fractional calculus [20,21]. We choose the M-L distribution as a tool to describe the distribution of fatigue data, since it has an apparent hereditary effect and power decay or heavy-tailed traits [22,23,24,25,26,27].

In this study, we will check the efficiency of the relative entropy method which is realized through the fractional order moment and the logarithmic moment by using the real experiment data of the fatigue life of the carbon fiber/epoxy composites instead of the produced random numbers using the Monte Carlo method. Then, we will study how to choose the best *p*-value in the fractional order moment algorithmic of the relative entropy method.

This paper is organized as follows. In Section 2, methodologies are provided. In Section 3, we test the feasibility of the relative entropy method and analyze the relationship between the level of stress and the efficient range of *p*. Finally, conclusions are drawn in Section 4. 

## 2. Criterion for Comparing

To measure the fatigue life of a composite, the relative entropy in the below form is used [17]:

(1) $$R\left(X_{dist}\right)=\left|\frac{H(X_{dist})-H(X_{em})}{H(X_{em})}\right|,$$

where H(∗) is the Shannon entropy of a random variable defined as

(2) $$\begin{array}{c} H\left(X\right)=-\sum_{i=1}^{n}P(X_{i})ln(P(X_{i}))\\ H\left(X\right)=-\int_{\Omega }ln(X)dF(X) \end{array}$$

For discrete and continues random variables, respectively. $H(X_{em})$ is the Shannon entropy of the experimental data, and $H(X_{dist})$ is the Shannon entropy of the distribution, which is fitted in terms of the experimental data. The candidates can be the M-L, Weibull and exponential distributions.

The relative entropy is valid according to the statistical theory [17,28,29], but it is a difficult problem for computing with a high cost to obtain the estimations of the probability density function and the Shannon entropy of the M-L distribution. In order to accelerate the speed with a lower cost, we often replace the Shannon entropy in Equation (1) by the fractional moment and the logarithmic moment. Actually, we calculate other quantities, but as criteria for specific usage, they perform consistently with the relative entropy [17,30,31]. In this sense, we call them approximations, as shown below.

Fractional moment approximation:

(3) $$R\left(X_{dist}\right)=\left|\frac{H(X_{dist}^{P})-H(X_{em}^{P})}{H(X_{em}^{P})}\right|,$$

And logarithmic moment approximation:

(4) $$R\left(X_{dist}\right)\approx \left|\frac{E(ln(X_{dist}))-E(ln(X_{em}))}{E(ln(X_{em}))}\right|.$$

The two methods were well justified by the preceding studies. There are also other candidates for fast calculation of the relative entropy which we leave out for convenience here.

In order to define the M-L distribution [17,30,31], we need two parameters, the stability index 0<α≤1 and the scale parameter σ≥0. If α=1, it degenerates into the classical exponential distribution, so it generalizes the exponential distribution. The M-L distribution is defined as

(5) $$f\left(x\right)=\frac{sin(\pi \alpha )}{\pi \sigma }\overset{\infty }{\underset{0}{\int }}\frac{y^{\alpha }e^{-\frac{xy}{\sigma }}}{y^{2\alpha }+2y^{\alpha }cos(\pi \alpha )+1}dy\;,$$

(6) $$F\left(x\right)=1-\frac{sin(\pi \alpha )}{\pi }\overset{\infty }{\underset{0}{\int }}\frac{y^{\alpha -1}e^{-\frac{xy}{\sigma }}}{y^{2\alpha }+2y^{\alpha }cos(\pi \alpha )+1}dy,x>0,$$

where f(x) is the probability density function (PDF), and F(x) is the cumulative distribution function (CDF).

The equivalence of the cumulative distribution function is in the form of series [17,30,31].

(7) $$F\left(x\right)=1-E_{\alpha }[-{\left(x/\sigma \right)}^{\alpha }],$$

(8) $$E_{\alpha }(x)=\sum_{k=0}^{\infty }\frac{x^{k}}{\Gamma (\alpha k+1)},$$

or

(9) $$F\left(x\right)=1-E_{\alpha ,\alpha }(-\frac{x^{\alpha }}{\sigma }),$$

(10) $$E_{\alpha ,\beta }(x)=\sum_{k=0}^{\infty }\frac{x^{k}}{\Gamma (\alpha k+\beta )}.$$

The characteristic function, i.e., the Laplace transform, is the same for Equations (6) and (7). More details of the relationship between Equations (6) and (7) can be found in [17,30,31].

The Weibull distribution is

(11) $$f=\frac{\alpha }{\beta }{\left(\frac{x-\varepsilon }{\beta }\right)}^{\alpha -1}e^{-{\left(\frac{x-\varepsilon }{\beta }\right)}^{\alpha }},$$

(12) $$F=1-e^{-{\left(\frac{x-\varepsilon }{\beta }\right)}^{\alpha }},x\geq \varepsilon .$$

The PDF and CDF of the exponential distribution are

(13) $$f(x)=\lambda e^{-\lambda x},$$

(14) $$F(x)=1-e^{-\lambda x}.$$

According to the former research, the relative entropy is better for the random variable with a spike PDF just as the M-L distribution is. Compared to the Weibull distribution and the exponential distribution through the Kolmogorov–Smirnov (K–S) test [17], the M-L distribution behaves much better to fit heavy-tailed data, such as the longevity and reliability of a composite suffering fatigue under different stress levels [17,30,31].

## 3. Data and Processing

The experimental data were from the experiments performed on an *AMSLER 10HFP1-478* fatigue test machine, and the tested specimens were carbon fiber/epoxy composites [32,33].

Firstly, we examined the fatigue life of the 50 axial tensile 480 MPa loading experiments [18,33]. The M-L distribution, the Weibull distribution and the exponential distribution were used to fit the empirical distribution of the experimental data. By fitting all these distributions, we obtained the parameters of the M-L distribution (α,σ)=(0.7, 2008400) with the method of the fractional moment estimation [30,31]; the parameters of the Weibull distribution (α,β,ε)= (0.51880, 2608231, 13097) with the least squares method (LSM); and the parameter of the exponential distribution $\lambda =\;4.9886\times 10^{6}$ with the least squares method (LSM).

Empirical entropy was calculated with the formula $E(ln(X_{em}))$, of which $X_{em}$ is the experimental data. Entropy of the M-L distribution was obtained by using the equation $E(ln(X_{ml}))=ln(\sigma )-\gamma$, where γ=0.5772 is the Euler’s constant. According to the preceding research [17], the fractional order moment is efficient when the parameter *p <* 0.4, so, firstly, we chose *p =* 0.2.

For any *0 < p < α*, the *p*-th moment of *X_ml_* has the expression $E(X_{ml}^{p})=\frac{p\sigma^{p}\pi }{\alpha \Gamma (1-p)sin(\pi p/\alpha )}$, where *α* is the stability index and *σ* is the scale parameter.

From Table 1 and Table 2, it is observed that the Weibull distribution is better than the M-L distribution, and the M-L distribution is better than the exponential distribution. The M-L distribution can be accepted to describe the experimental data. It is different from the K–S test, in which the M-L distribution is the best [18]. However, it is consistent with the widely accepted conclusion that Weibull distribution is superior to other distributions. It should be pointed out that based on the existing results given in [18], for the tail part of the distribution, the M-L distribution is the best.

**Figure 1:**
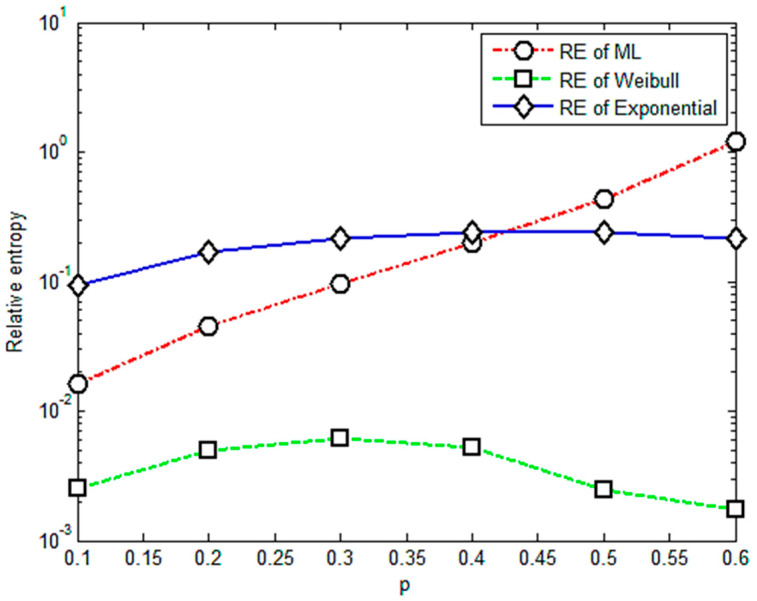
Relative entropy versus variable orders of the fractional order moment.

To choose the proper value of *p* to improve the effectiveness and reliability of the relative entropy method, we changed values of *p* from 0.1 to 0.6. The values of the parameters *p* should be smaller than α=0.7 in this case, which is also consistent with the case in [17]. Figure 1 gives the results for the relative entropy with varied values of *p* from 0.1 to 0.6 based on the experiment data for an axial tensile multi-amplitude loading.

**Figure 2:**
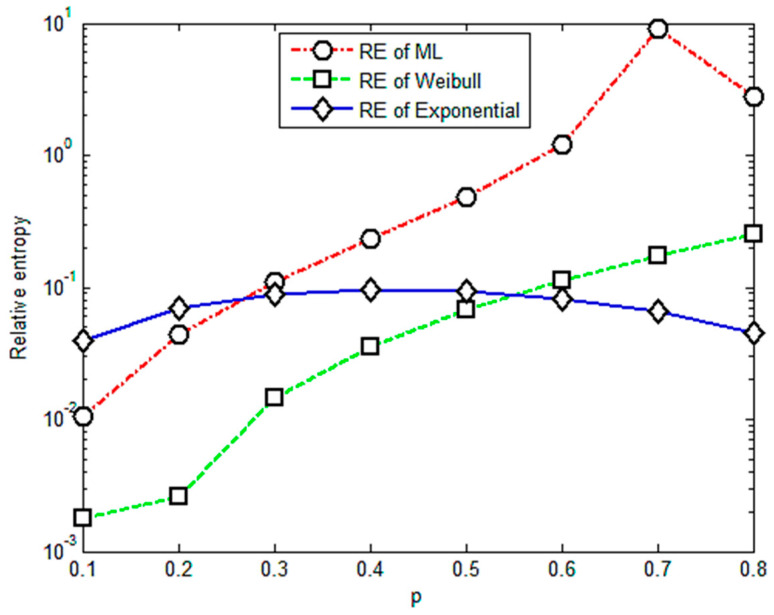
Relative entropy versus variable orders of the fractional order moment under stress of 471 Mpa.

**Figure 3:**
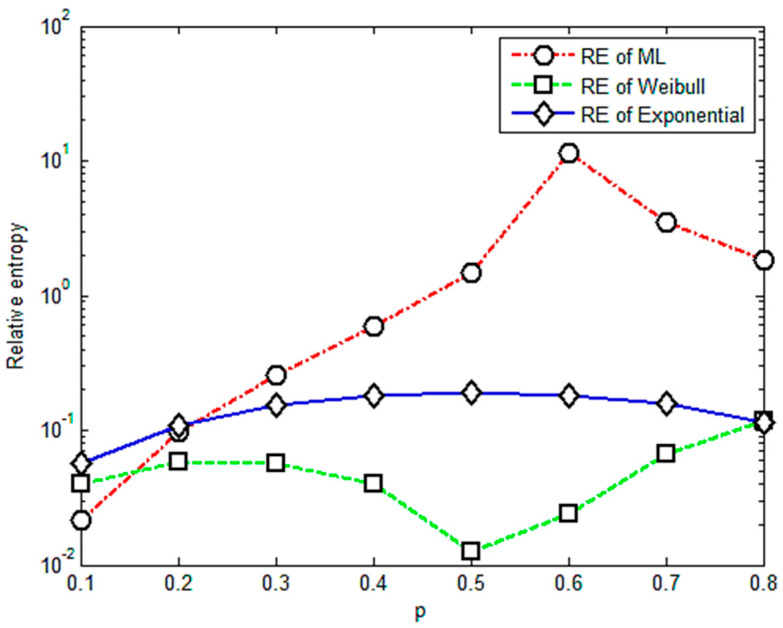
Relative entropy versus variable orders of the fractional order moment under stress of 530 Mpa.

**Figure 4:**
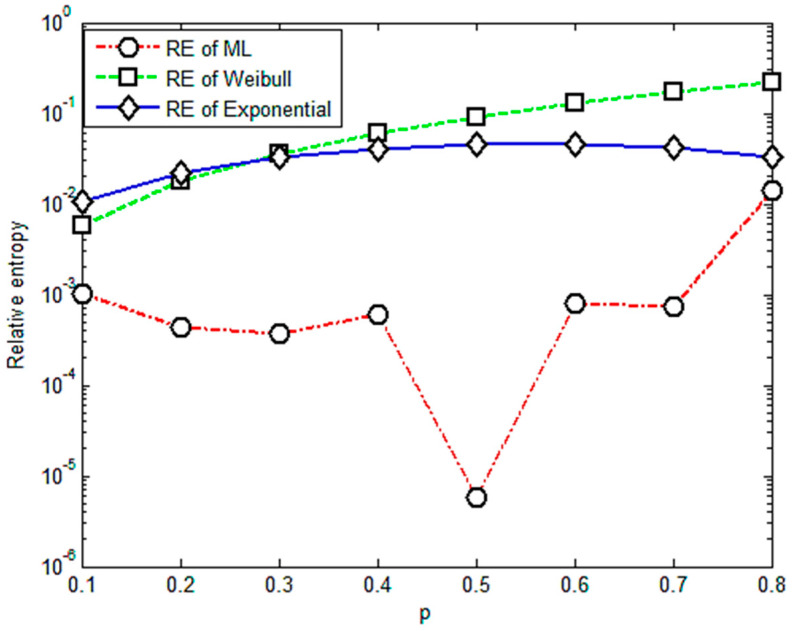
Relative entropy versus variable orders of the fractional order moment under stress of 588 Mpa.

**Figure 5:**
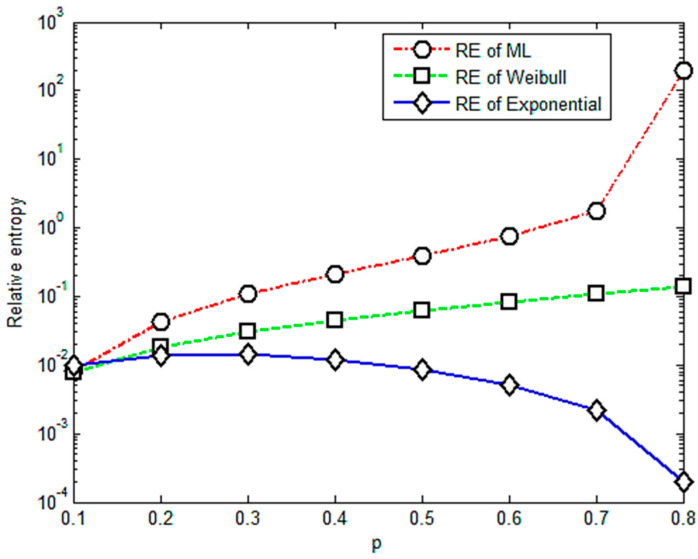
Relative entropy versus variable orders of the fractional order moment under stress of 667 Mpa.

It is apparent that Figure 1 shows an almost positive correlation between relative entropy and the value of *p*. The relative entropies of the M-L and Weibull distributions are found smaller than those of the exponential distribution when *p* is less than 0.4, illustrating that the fractional order moment is valid. The value of parameter *p* is acceptable if it is less than 0.4 or its corresponding relative entropy is less than 0.3. Based on this criterion, it is concluded that under multi-amplitude loading, the fractional order moment is efficient when the parameter *p <* 0.4. With more data for different levels of stress, the corresponding results are given in Figure 2, Figure 3, Figure 4 and Figure 5.

Figure 2 shows that the relative entropies of the M-L and Weibull distributions are smaller than the exponential distribution and there is a positive correlation between relative entropy and the value of *p* when *p* is less than 0.3; therefore, the upper limit of the effective range of the value of *p* is 0.3.

Figure 3 shows that the relative entropies of the M-L and Weibull distributions are smaller than the exponential distribution and there is a positive correlation between relative entropy and the value of *p* when *p* is less than 0.2; thus, the upper limit of the effective range of the value of *p* is 0.2.

Figure 4 shows that relative entropies of the M-L and Weibull distributions are smaller than the exponential distribution and M-L is better than Weibull when *p* is less than 0.3, so the upper limit of the effective range of the value of *p* is 0.3.

Figure 5 shows that relative entropies of the M-L and Weibull distributions are always bigger than the exponential distribution. Therefore, the relative entropy method is not valid for these group data since its result does not agree with the widely accepted one. Another point should be mentioned that there are drops in Figure 3 and Figure 4 for the case of *p* = 0.5. The probable reasons for this point are the variation and randomness of the experimental data. We will use more data and try different composite materials in further study to detect the underlying physical law of these points.

## 4. Conclusions

With the foregoing results, we can draw the following conclusions:

1. The relative entropy method is not fit for choosing the proper distribution for non-exponential random data if we want to emphasize the heavy tail trait of the data. It is not consistent with the K–S test.
2. The relative entropy is consistent with other indexes such as the residual sum of squares in the least squares method whenever it is calculated by the fractional moment or the logarithmic moment.
3. Under different stress amplitudes, the relative entropy method has different performances.
4. The relative entropy can be considered the first step to determine the candidates of the fatigue distribution and is feasible to select the suitable fatigue distribution of the fatigue life, which does not follow the exponential distribution.

## Figures and Tables

**Table 1:** Entropy and relative entropy calculated by using the logarithmic moment.

Distribution	M-L	Weibull	Exponential	Empirical
Entropy	13.9356	13.8462	14.8455	13.826
Relative entropy	0.0079	0.0015	0.0737	0

**Table 2:** Entropy and relative entropy calculated by using the fractional order moment (*p =* 0.2).

Distribution	M-L	Weibull	Exponential	Empirical
Entropy	17.968	17.2821	20.0687	17.1961
Relative entropy	0.0449	0.005	0.167	0

## Data Availability

The data presented in this study are openly available at [doi:10.19515/j.cnki.1003-1545.1994.04.004], Reference number [33].
